# The Role of Dipeptidyl Peptidase – 4 Inhibitors in Diabetic Kidney Disease

**DOI:** 10.3389/fimmu.2015.00443

**Published:** 2015-08-28

**Authors:** Usha Panchapakesan, Carol Pollock

**Affiliations:** ^1^Renal Research Group, Kolling Institute of Medical Research, Royal North Shore Hospital, University of Sydney, Sydney, NSW, Australia

**Keywords:** dipeptidyl peptidase 4, diabetic kidney disease, fibrosis, inflammation, renoprotection

## Abstract

Despite major advances in the understanding of the molecular mechanisms that underpin the development of diabetic kidney disease, current best practice still leaves a significant proportion of patients with end-stage kidney disease requiring renal replacement therapy. This is on a background of an increasing diabetes epidemic worldwide. Although kidney failure is a major cause of morbidity the main cause of death remains cardiovascular in nature. Hence, diabetic therapies which are both “cardio-renal” protective seem the logical way forward. In this review, we discuss the dipeptidyl peptidase 4 (DPP4) inhibitors (DPP4inh), which are glucose-lowering agents used clinically and their role in diabetic kidney disease with specific focus on renoprotection and surrogate markers of cardiovascular disease. We highlight the novel pleiotropic effects of DPP4 that make it an attractive additional target to combat the fibrotic and inflammatory pathways in diabetic kidney disease and also discuss the current literature on the cardiovascular safety profile of DPP4inh. Clearly, these observed renoprotective effects will need to be confirmed by clinical trials to determine whether they translate into beneficial effects to patients with diabetes.

## The Incretin System

Under physiological conditions, blood glucose is tightly regulated by a number of mechanisms including the incretin system of hormones that are secreted in response to a meal. Glucagon like peptide 1 (GLP-1) and gastrointestinal peptide (GIP) are released from the gut and regulate postprandial glucose excursions. Proglucagon is secreted by the intestinal mucosa and is subsequently cleaved to GLP-1, which acts on the GLP-1 receptor in the pancreas in a glucose-dependent manner and functions to stimulate pancreatic insulin release and suppress glucagon secretion with the net effect of regulating postprandial glucose (1). The half-life of GLP-1 is very short (minutes) as it is rapidly degraded by dipeptidyl-peptidase 4 (DPP4). DPP4 cleaves two amino acids at the amino terminal end of peptides that have a proline (or less commonly an alanine) at the penultimate position (2). Hence GLP-1 (7–36) amide is cleaved to GLP-1 (9–36) amide, which is the major circulating form. GLP-1 (9–36) does not have insulinotropic effects.

Dipeptidyl peptidase 4 inhibitors (DPP4inh) are novel oral diabetic agents used to lower blood glucose in patients with type 2 diabetes mellitus. They inhibit DPP4, which degrades GLP-1 resulting in raised endogenous GLP-1. They promote insulin release and inhibit glucagon secretion, causing lower blood glucose. Favorable aspects of DPP4inh in comparison to other diabetic agents include its oral formulation, reduced risk of hypoglycemia and lack of weight gain. In addition, DPP4inh have benefits beyond glucose lowering as DPP4 cleaves a host of peptides/substrates in addition to GLP-1, resulting in a broad range of altered biological functions.

**Figure 1:**
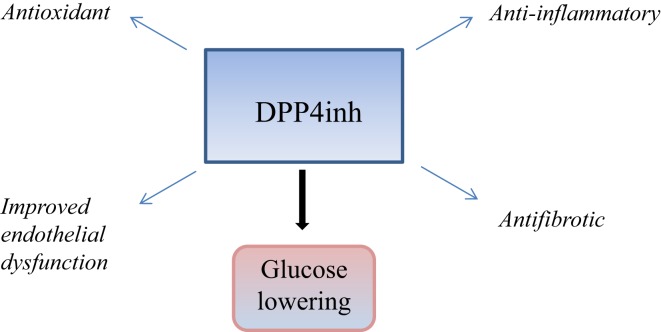
**This is a schematic diagram summarizing the glucose lowering and pleiotropic effects of DPP4 inhibition, which may be relevant in cardiorenal protection**.

In this review, we focus on the pleiotropic properties of DPP4 beyond glucose lowering with specific reference to renoprotection and cardiovascular aspects. This is summarized in Figure 1. The clinical relevance of this is the fact that up to a third of patients with diabetes suffers renal complications with the main cause of mortality being cardiovascular in origin. Hence, continued inquiry into mechanisms of renal failure in patients with diabetes that will lead to examination of novel strategies to limit cardiovascular and renal disease is the logical step forward.

## DPP4 – A Multifunctional Protein

DPP4 is a 110-kDa type 11 integral membrane glycoprotein and is expressed ubiquitously in most organs and cell types. DPP4 exists in both a soluble and membrane bound form, both of which are capable of proteolytic activity. The soluble form in the circulation is thought to arise from shedding of the membrane bound DPP4 and is the target for DPP4inh as hypoglycemic agents in clinical use (1). Identified substrates include GLP-1, regulated on activation, normal T cell expressed and secreted (RANTES), which is relevant in renal disease (3, 4), brain natriuretic peptide 1–32 (5, 6), neuropeptide Y (7), high mobility group protein 1 (HMGB1) (8), and Substance P (9). More recently, aggregates of amyloid beta peptides (pathological hallmarks of Alzheimers disease) have also been identified as substrates of DPP4 (10). Many substrates have been identified as being pharmacological substrates (cleaved *in vitro*) but are not cleaved *in vivo*. Hence, this proteolytic effect can either lead to a gain in function, a loss of function or have no functional significance. An example of the physiological relevance of these non-GLP-1 substrates is highlighted by DPP4 truncation of certain chemokines, colony-stimulating factors, and interleukins, which have recently been linked to regulation of hematopoietic stem/progenitor cells where DPP4inh has been shown to enhance engraftment of cord blood in patients with hematological malignancies (11, 12).

The membrane bound form of DPP4, expressed on the surface of many cell types including kidney tubular cells, endothelial cells and T cells, is of major interest with respect to the pleiotropic actions of DPP4. Membrane-bound DPP4 also exerts non-enzymatic actions by virtue of co-localizing with other membrane proteins and modulating their intrinsic actions (2, 13). DPP4 is known to interact with adenosine deaminase (14), caveolin 1 (15, 16), cation independent mannose 6 phosphate receptor (CIM6PR) (also known as insulin growth factor receptor 2) (17, 18), and glypican 3 (19). Interestingly, it has been recognized that DPP4 interacts with human coronavirus-EMC spike protein and acts as a receptor for this virus, which provides new knowledge to facilitate the direction of interventional strategies in related respiratory illness (20).

## DPP4 and the Kidney

The proteolytic functions of DPP4 in the kidney have been described using liquid chromatography mass spectrometry-based peptidomics, where kidney tissue from DPP4^+/+^ and DPP4^−/−^ mice were compared. This revealed 10 peptides regulated by DPP4 *in vivo* (21, 22). Further studies with brush border membranes showed that aminopeptidase activity is required to generate DPP4 substrates. This suggests that DPP4 is involved in the extracellular catabolism of proteins in the kidney, specifically the degradation/catabolism of proline-containing peptides (23). As DPP4 is present on the brush border (apical side) of kidney proximal tubular cells, DPP4 inhibition is likely to alter the degradation/regulation of peptides in the lumen and thus influence the tubular cell structure or function in diabetes. Identifying the functionally relevant renal substrates of DPP4 will help us understand and anticipate long-term effects of DPP4 inhibition on the kidney in patients with diabetes. The tools required to validate identified substrates are limited given that specific antibodies must be able to distinguish between cleaved and non-cleaved peptides, which differ by only two amino acids at the N-terminal end.

Transforming growth factor beta 1 (TGFβ1) is a major driver of fibrosis in diabetic kidney disease. We have reported that linagliptin, a DPP4inh, reduces high glucose-induced active TGFβ1 in human kidney proximal tubular cells (24) with a downstream reduction in phosphorylated smad2 and fibronectin transcription and expression. As high-glucose-induced total secreted TGFβ1 was unchanged by linagliptin, we postulated that the mechanism was related to interference with the conversion from latent to active TGFβ1. TGFβ1 is secreted in a latent form and requires a complex interplay of soluble signaling molecules in the activation process, which releases it from the latency associated peptide (LAP) (25). Once released from the LAP, the unbound TGFβ1 can then bind to its receptor to initiate cell signaling via the Smad pathway. In pursuing this further, we showed that that the interaction between CIM6PR and DPP4 is “switched on” by high glucose, and hence is maximally modulated by linagliptin in this environment. In the presence of excess M6P, the CIM6PR binding sites become saturated, resulting in the reduction in CIM6PR/DPP4 interaction, which would imply that the interaction is occurring through a M6P residue on the DPP4 molecule. The fact that linagliptin also reduced this interaction, suggests a M6P-mediated mechanism, which is independent of GLP-1 as our *in vitro* system is lacking in GLP-1 (unpublished data). This novel finding adds to the body of knowledge that DPP4 inhibition can exert antifibrotic effects through its non-proteolytic properties. This is discussed further below with respect to endothelial cell dysfunction.

In interpreting preclinical studies, it is important to appreciate the confounding aspects of concomitant glucose lowering by DPP4inh. There are two studies, which have looked at the effect of DPP4inh (using vildagliptin and sitagliptin) on the diabetic animal kidney and both show renoprotection. However, the HbA1c (hemoglobin A1C) in the DPP4inh-treated diabetic animals was lower than in the diabetic-control animals (26, 27). So in both these *in vivo* studies, it is difficult to conclude that the renal effects of DPP4inh lie above and beyond glucose lowering.

Kanasaki and colleagues investigate the antifibrotic properties of linagliptin in a Type 1 model of diabetic nephropathy and showed that after 4 weeks, linagliptin ameliorated diabetic kidney fibrosis, an observation that occurred in association with the inhibition of endothelial-mesenchymal transition (EndMT) and the restoration of microRNA29s (28). An insulin-deficient model of diabetes enables evaluation of the effects of the DPP4inh independent of glycemic control as DPP4inh will not have a significant hypoglycemic effect due to the lack of a significant insulin response mediated by GLP-1. As a result, the new findings provide information regarding renal benefit of linagliptin independent of glycemic control.

The antifibrotic properties of DPP4inh have also been shown in other models of kidney fibrosis such as the unilateral ureteral obstruction (UUO) model (29). In this TGFβ-driven model, LC15-0444 (a DPP4inh) reduced inflammatory and fibrotic markers such as levels of phosphorylated Smad2/3, TGFβ1, toll-like receptor 4, HMGB1, NADPH oxidase 4, and nuclear factor kappa B. However, this study lacked specific mechanistic data to explain the findings.

There is also experimental evidence to suggest that DPP4 is involved in the advanced glycation end product – receptor axis, which is highly relevant in diabetic kidney disease. Both DPP4 deficiency and linagliptin reduced renal damage in animal models of Type 1 diabetic nephropathy (30, 31). The mechanism proposed was related to reducing endothelial cell oxidative stress.

Likewise, DPP4inh has been explored in animal models of obesity, insulin resistance, and renal disease. The DPP4inh MK0626 was shown to reduce obesity-induced renal injury (MK0626) and attenuated filtration barrier injury and oxidant stress in the zucker obese rat (linagliptin) (32, 33).

Endothelial dysfunction is an inherent aspect of diabetic kidney disease. Shi et al. demonstrated that in endothelial cells, DPP4 and integrin β1 physically interacted. DPP4-associated endothelial to mesenchymal transition was inhibited by integrin β1 deletion. In addition, DPP4 or integrin β1 deficiency resulted in the inhibition of TGFβ2-stimulated heterodimer formation of TGFβ receptors. Along with this, the interaction between DPP4 and integrin β1 induced vascular endothelial growth factor receptor (VEGF-R) 1 expression with the concomitant suppression of VEGF-R2 levels (34). Importantly, linagliptin was able to reduce the TGFβ2 physical interaction between integrin and DPP4. These results indicate that the non-proteolytic interaction between DPP4 and integrin β1 is relevant in diabetic kidney fibrosis.

Mechanistic studies designed to delineate whether effects on glucose-induced endothelial impairment are related or independent of GLP-1/GLP-1R showed that in addition to GLP-1 receptor effects, DPP4inh with linagliptin exerted direct effects on rat mesenteric vessels independent of any glucose lowering effect. The mechanism was shown to be related to its antioxidant effect through direct radical scavenging (35).

DPP4 inhibitors also exert anti-inflammatory effects. This has been demonstrated in a non-diabetic animal model of glomerular injury where alogliptin reduced macrophage infiltration (36) and likewise in a type 1 diabetic nephropathy model where PKF275-055 reduced macrophage infiltration and nuclear factor kappa B activity (37).

It is increasingly evident that although DPP4inh have similar capacity to improve glucose control they have very different pharmacokinetic and pharmacodynamic profiles as they bind to different sites on DPP4 (38). All DPP4 inhibitors can block the enzymatic (i.e., cleaving) property of DPP4 but may differ in their ability to influence the co-stimulatory/receptor properties of DPP4 (2). The mode of clearance of DPP4 inhibitors may also be relevant (39). DPP4 is highly expressed on the luminal aspect of the proximal tubular brush border which implies that drugs which are preferentially filtered by the kidney have better access to tubular DPP4.

In summary, preclinical studies suggests that DPP4inh offer renoprotection above and beyond glucose lowering through its proteolytic, protein–protein interaction, and antioxidant properties. Given the increased cardiovascular risk in this population, it would be highly desirable to have a glucose-lowering drug, which also offers cardiovascular benefit in addition to renoprotection. Regulatory agencies now mandate that new diabetic agents undergo cardiovascular assessment prior to marketing. The agreed-on primary endpoint for cardiovascular safety analyses is Major Adverse Cardiovascular Events Plus (MACE-plus), defined as a composite endpoint consisting of the following adjudicated events: cardiovascular death, non-fatal myocardial infarction, non-fatal stroke, and hospitalization for unstable angina (40). The next section summarizes major clinical trials in this area with cardiovascular and renal endpoints.

## Cardiovascular Aspects

Clinical trials evaluating both cardiovascular and renal endpoints have commenced with the CARMELINA study, which will include more than 8,000 adults with Type 2 Diabetes and linagliptin (ClinicalTrials.gov Identifier: NCT01897532). The primary endpoint will be time to the first occurrence of either cardiovascular death (including fatal stroke and fatal myocardial infarction); non-fatal myocardial infarction; non-fatal stroke; or hospitalization for unstable angina pectoris. The renal outcome will be measured as a composite of renal death, sustained end-stage renal disease and sustained decrease of ≥50% eGFR.

Although DPP4inh have offered some promise with pre-clinical studies and *post hoc* analyses of clinical trials suggesting a positive cardiovascular risk profile, a recent randomized placebo controlled trial (SAVOR-TIMI 53) published in the New England Journal of Medicine by Scirica et al. showed no change in ischemic events but higher hospitalizations for heart failure with saxagliptin. This study enrolled 16,492 patients with type 2 diabetes with a history of, or were at risk for, cardiovascular events and followed up for a median of 2.1 years (41). The EXAMINE study evaluated alogliptin use in patients with type 2 diabetes after acute coronary syndromes showed that there was no increase in the rates of MACE-plus (42). This randomized placebo-controlled trial involved 5,380 patients and had a median follow up of 18 months. The US Food and Drug Administration (FDA) independent advisory committee recently voted that the cardiovascular safety profile of these two drugs could be managed by updating the drugs’ labels (43). Recently, a large multicentre clinical trial TECOS (Sitagliptin on Cardiovascular Outcome Study) where 14,671 patients with established cardiovascular disease and Type 2 diabetes were randomized to receive either saxagliptin or placebo (in addition to their usual therapy) showed no increase in the risk of MACE-plus, hospitalization for heart failure, or other adverse events (44). Another large multicentre trial with linagliptin CAROLINA (Cardiovascular Outcome Study of Linagliptin Versus Glimepiride in Patients with Type 2 Diabetes) is currently in progress (44). As DPP4inh raise GLP-1, it is also important to take into account the cardiovascular profile of GLP-1 analogs. In a large retrospective analysis by Best et al., patients treated with the GLP1 analog exenatide were shown to have a 20% reduction in cardiovascular events in comparison with other glucose-lowering agents (45). However, the studies that have demonstrated cardiovascular benefit using GLP1 or an analog do so using supra physiological doses. DPP4inh promote GLP1 action only within the physiological range and DPP4inh are likely to have pleiotropic effects independent of their effects on GLP1 as outlined above. Further details about these trials can be found in www.clinitrials.gov. There are several DPP4 inhibitors in clinical use e.g., sitagliptin, saxagliptin, vildagliptin, alogliptin and linagliptin. They differ structurally and in their pharmacokinetic profiles and hence the outcome of ongoing trials is important as a class effect cannot be presumed.

In conclusion, DPP4 inhibitors hold promise as an antifibrotic and anti-inflammatory agent in kidney disease with a large clinical trial currently underway to evaluate hard renal endpoints. The jury is still out on whether the risk of heart failure is a class effect.

## Conflict of Interest Statement

Usha Panchapakesan and Carol Pollock have received linagliptin and funding from Boehringer Ingelheim Germany as well as saxagliptin and funding from Bristol Myer Squibb.
